# Catalyst-Support Interactions Promoted Acidic Electrochemical Oxygen Evolution Catalysis: A Mini Review

**DOI:** 10.3390/molecules28052262

**Published:** 2023-02-28

**Authors:** Zijie Luo, Jia Wang, Wei Zhou, Junsheng Li

**Affiliations:** 1 School of Chemistry, Chemical Engineering and Life Sciences, Wuhan University of Technology, Wuhan 430070, China; 2 School of Science, Wuhan University of Technology, Wuhan 430070, China

**Keywords:** fuel cell, water electrolysis, catalyst support interactions, oxygen evolution

## Abstract

In the context of the growing human demand for green secondary energy sources, proton-exchange membrane water electrolysis (PEMWE) is necessary to meet the high-efficiency production of high-purity hydrogen required for proton-exchange membrane fuel cells (PEMFCs). The development of stable, efficient, and low-cost oxygen evolution reaction (OER) catalysts is key to promoting the large-scale application of hydrogen production by PEMWE. At present, precious metals remain irreplaceable in acidic OER catalysis, and loading the support body with precious metal components is undoubtedly an effective strategy to reduce costs. In this review, we will discuss the unique role of common catalyst-support interactions such as Metal-Support Interactions (MSIs), Strong Metal-Support Interactions (SMSIs), Strong Oxide-Support Interactions (SOSIs), and Electron-Metal-Support Interactions (EMSIs) in modulating catalyst structure and performance, thereby promoting the development of high-performance, high-stability, low-cost noble metal-based acidic OER catalysts.

## 1. Introduction

Due to the non-renewable nature of fossil fuels, excessive consumption of fossil fuels for energy is bound to cause serious ecological problems [1]. According to a report by the International Energy Agency (IEA), total global energy demand will exceed the equivalent of 18 billion tonnes of oil by 2030 [2]. Therefore, the development of secondary energy is crucial to meet the needs of sustainable development. The need for efficient production of renewable, non-polluting secondary energy is also urgent to meet people’s quest for a higher standard of living while preserving the ecological environment. Recently, renewable energy systems such as solar power, wind and tidal energy are considered as potential green secondary energy. However, the large-scale application of these energy conversion devices has been hindered by the limitations of their geographical nature, periodicity and high cost of long-distance transmission [3,4,5].

As a power generation device, proton-exchange membrane fuel cells (PEMFCs) have attracted various attention. PEMFCs can efficiently convert the chemical energy of hydrogen and oxygen directly into electrical energy [6]. On the other hand, PEMFCs have lower energy losses and higher energy conversion efficiencies because they do not need to go through the Carnot cycle to convert energy [7]. On the other hand, PEMFCs also have the advantage of zero missions, as the only running product is pure water. The raw material required for the PEMFCs is high-purity hydrogen. Oetjen et al. found that for Pt-based catalysts in PEMFCs, even if exposed to feed gas containing 100 ppm CO for 5 min, the catalytic performance would significantly decline [8]. Currently, conventional hydrogen production processes are still based on natural gas reforming. However, hydrogen obtained through the natural gas reforming process could not meet the standard required for PEMFCs application, due to its poor purity [2,9]. It would also be impractical to purify the hydrogen, which would add to the cost of production. The purity of hydrogen produced by proton-exchange membrane water electrolysis (PEMWE) is up to 99.99%, which has obvious advantages over other hydrogen production methods [10]. Therefore, for PEMFCs to be deployed on a large scale, the promotion of PEMWE is of great importance.

The hydrogen produced by PEMWE is an ideal source of feed gas for the large-scale production of PEMFCs. PEMWE has the advantages of compact design, small footprint, high current density [11,12]. In practice, noble metals like Ir and Ru catalysts are heavily used in the OER catalysts designed to lower the potential barrier, leading to the high cost of PEMWE. According to the report, the cost of hydrogen production with PEMWE is as high as ~$10.30/kg, and only ~4% of the hydrogen is produced through PEMWE, due to the high cost [13]. Therefore, it is of great significance to design cost-effective, efficient and highly stable OER catalysts.

As OER is a multi-phase catalytic process, the substances that surround the active component have a strong influence on the catalytic process. This has been proved in recent years by many studies of loaded catalysts for multi-phase catalysis. Yin et al. reported a novel nitrogen-doped ordered mesoporous carbon-supported Pt (Pt@NOMC-A) catalyst with a low Pt loading of 7.2 wt%, and showed that the synergy between Pt nanoparticles (NPs) and carbon support, as well as the physical confinement offered by the carbon support, enhanced the electrochemical performance of the novel catalyst. Pt@NOMC-A exhibits a low hydrogen evolution reaction (HER) overpotential, comparable with commercial 20 wt% Pt/C catalyst under acidic, neutral and alkaline conditions [14]. Li et al. reported that by introducing synergistic interaction between Pt and substrate material (NiS_2_), the favorable synergy interaction can modify the d-band structure of the Pt facet (111) and modulate the hydrogen adsorption on Pt (111), which enhances the intrinsic electrocatalytic activity of Pt [15]. Wei et al. proposed a strategy to enhance the performance of ENRR catalysts by catalyst-support interactions. Both experimental and theoretical investigations show that the electron is transferred from ZrO_2_ substrate to Au. Such an electron transfer process modifies the electronic structure of Au, which enhances the ENRR activity HER performance of the Au active components [16]. In the WO_3_/ZrO_2_ solid acid electrocatalyst for alkaline HER reported by Wei et al., the acidity of the electrocatalyst is controlled by the amount of WO3 loaded on the ZrO2. Experimental analysis demonstrated that catalyst-support interactions in WO3/ZrO2 can modulate the acidity of the electrocatalyst and thus reduce the hydrolysis potential barrier [17]. Ma et al. have reported a large-scale synthesis method for hollow and microporous/mesoporous nitrogen-doped carbon capsules functionalized with single-atom iron (FeN_4_) sites in the walls. Aided by the catalyst-support interaction between the FeN_4_ site and the graphite domain substrate, the single-Atom iron is well-dispersed in the N-doped carbon support. Benefiting from its open porous structure and the accessibility of the FeN_4_ sites, H-Fe-Nx-C showed excellent ORR activity and long-term stability, surpassing commercial Pt/C [18]. The Co-SAC/NC reported by Rao et al., due to the well-dispersed single Co atoms on the support, which gives a robust ORR performance in an alkaline medium [19]. The Co_3_O_4_/NiO_x_H_y_ reported by Wang et al. has facilitated the OER kinetics at the interface in a completely different way, by means of catalyst-support interactions [20]. These works demonstrate the potential of catalyst-support interactions to modulate catalyst activity, which has implications for the development of efficient OER catalysts.

Herein, we will discuss the unique role of common catalyst-support interactions such as metal-support interactions (MSI), strong metal-support interactions (SMSI), strong oxide-support interactions (SOSI), and electron-metal-support interactions (EMSI) to provide ideas for the development of novel acidic OER catalysts.

## 2. Fundamentals and Current Status of Research on Acidic OER

**Figure 1:**
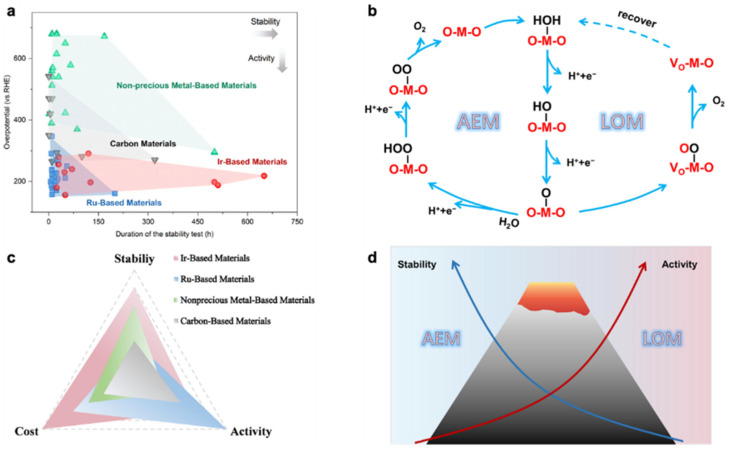
(**a**) Stability test time and ŋ10 of catalysts reported in recent years and (**b**) acid OER reaction mechanism. (**c**) The stability, activity, and cost of Ir-, Ru-, nonprecious metal-, and carbon-based materials. (**d**) Schematic diagram of the difference in stability and activity of the AEM/LOM mechanism [24].

Although the O_2_ produced by the OER reaction is a by-product of the electrolysis of water for hydrogen production, the reaction kinetics are slower in an acidic environment due to the fact that OER is a four-electron process and requires higher energy to overcome the high potential barrier of the kinetics [21]. As a result, the anode reaction consumes the most energy process in the entire electrolytic water process. In order to reduce the overpotential, large amounts of precious metal are used in the anode catalysts. Ir-based and Ru-based catalysts exhibit impressive catalytic activity for OER reactions in acidic media [22]. It is now generally accepted that the acidic OER process is dominated by two mechanistic routes, the adsorbed evolution mechanism (AEM) and lattice oxygen mechanism (LOM) [23]. As shown in Figure 1b, the OER process through AEM involves four steps and proceeds on a single adsorption site. There is a linear relationship between the adsorption energies of O*, *OH and *OOH [24]. Utilizing isotopic labelling, researchers have investigated the contribution of lattice oxygen to OER. The results show that the acidic OER process is co-dominated by both AEM and LOM. The proportion of LOM is related to the catalyst structure [25,26]. The LOM process typically has a lower activation barrier than the AEM process. However, the LOM process can lead to degradation of the active component of the catalyst, resulting in reduced stability of the OER catalyst [23,27].

Ir and Ru are noble metals, which is the main cost of electrolytic cells. This has become the biggest obstacle to the industrialization of PEMWE. However, in acidic media and under high-voltage conditions, both Ru-based and Ir-based catalysts are not sufficiently stable [28,29,30], which is not sufficient for the large-scale application of PEMWE. Great efforts have been made to improve the atomic utilization of precious metals and to enhance the performance and stability of acidic OER catalysts. Ru/Mo_2_C heterojunctions were constructed to regulate the valence of Ru and Mo in the catalyst to simultaneously boost OER and HER in acidic conditions [31]. A chemical etching strategy for fabricating a Ru/Fe oxide towards OER resulted in enhanced stability [32]. Sr–Ru–Ir ternary oxide electrocatalysts: The electronic structure of active Ru sites was modulated by Sr and Ir, resulting in an overpotential of 190 mV at 10 mA cm^−2^, and the overpotential remained below 225 mV following 1500 h of operation [30]. Rh-Ir alloy NPs prepared by a scalable microwave-assisted method with improved atom utilisation through ~10 nm ultra-fine size: Owing to the synergy of ensemble and electronic effects by alloying a small amount of Rh with Ir, the binding energy difference of the O and OOH intermediates was reduced, leading to faster kinetics and enhanced OER activity [33]. 

It has also been shown that alloys [34,35,36], perovskite material [37,38,39,40], single-atomic materials [41,42,43,44], MOF materials [45,46,47,48], and transition metal oxides [49,50] have potential applications in acidic OER. Unfortunately, as is shown in Figure 1c, due to the harsh acidic conditions, it seems difficult to accomplish the balance of stability and catalytic performance with these reported non-precious metal catalysts. Therefore, the current position of noble metal catalysts in OER catalysts is still unshakable. Due to the high cost of noble metals, improving catalytic activity and durability of catalysts while reducing the amount of noble metals is the focus of current research. To further improve the industrial viability of PEMWE, researchers have explored many strategies that can significantly enhance the catalytic activity and stability of Ru-based and Ir-based catalysts for OER in acidic media, such as doping [51,52,53], morphological engineering [54,55], defect engineering [56,57], etc. Among the many catalyst modification methods, dispersing and loading the noble metal component of the catalyst onto the support is undoubtedly a viable strategy to reduce the noble metal loading and enhance the atomic utilization. It is found that the interaction between the catalyst and the support at the nanoscale can improve the catalytic performance and enhance the durability of the catalyst to a certain extent [57]. Therefore, the interaction between catalysts and supports has attracted the attention of more researchers in recent years. Meanwhile, probing the nature of the interaction of catalyst-support interface structure at the atomic scale is crucial to enhance the catalytic performance of acidic OER catalysts and to advance the industrialization of PEMWE. In the following, we will compile and summarize common catalyst-support interactions in supported acidic OER electrocatalysts.

## 3. Support-Catalyst Interactions in Acidic OER

Supported catalysts are widely used in the fine chemical industry and different energy conversion processes. These catalysts are generally prepared by loading the active component onto the surface of solid phase support. The activity and selectivity of the catalyst are controlled by regulating the particle size, morphology and composition of the active component [58]. Initially, it was thought that the main role of the support was to disperse and stabilize the active metal components and to improve the mechanical strength and heat resistance of the catalyst. However, as research progressed, the physical and chemical interactions between the active component and the support were found to have a significant impact on the performance of the catalyst [58]. Currently, the role of solid-phase supports includes: (1) effectively dispersing metal NPs and improving utilization of catalytic active sites; (2) provision of an effective channel for the diffusion of reactants for the enhancement of the reaction rate; (3) acting as a medium for heat transfer and electron transport. Supports for acidic OER catalysts, such as transition metal oxides [59], metal carbides [60], and carbon-based materials [61], have been studied recently. The design of OER catalysts with strong catalyst-support interactions guided by theoretical calculations is considered a powerful strategy to achieve higher precious metal utilization. Currently, there are four main types of catalyst-support interactions: metal-support interaction (MSI), strong metal-support interaction (SMSI), strong oxide-support interaction (SOSI), and electron-metal-support interaction (EMSI).

### 3.1. Metal Support Interaction (MSI)

MSI is commonly found in supported metal-based catalysts. Metal NPs are a fundamental component of various catalysts due to their unique electronic structure, morphology and controlled composition. Immobilization of metal NPs on support could improve their stability and control their spatial distribution. In addition, since carriers are usually not inert, the interaction between carriers and metal NPs can generate specific interfacial phenomena [62]. The main phenomenon in the catalytic material induced by MSI is fourfold as follows.

#### 3.1.1. Charge Transfer

Due to the difference in Fermi energy levels between metal NPs and supports, interfacial contact between NPs and supports can induce changes in the electronic structure of both supports and NPs to seek an equilibrium in the electrochemical potential, and such changes are bi-directional in nature [63]. The metallic properties of the NPs give the electrons mobility. However, the mobility is related to the nanosystem because the smaller the NP, the more localized its electronic state is. In some cases, the charge transfer may be accompanied by a change in the oxidation state of the NPs or the metal ions of the supports [64]. Qin et al. synthesized a core-shell nanoparticle (Ru@Ir-O) with an Ru core and an oxygen-bound Ir shell. With an OER overpotential as low as 238 mV at a current density of 10 mA cm^−2^, a high mass activity of 1169.0 A g^−1^ was achieved at 1.55 V, which was five and 78 times higher than that of pure Ir and IrO_2_, respectively. Structural characterization and density functional theory (DFT) calculations show that MSI and tensile strain between the core-shell led to charge redistribution, optimizing the bonding strength of O* and HOO* intermediates on the catalyst surface, which in turn improved electrocatalytic OER activity. This work not only provides a feasible method for the synthesis of efficient core-shell nanocatalysts, but also demonstrates an effective strategy to modulate OER performance through the charge transfer effect of MSI in concert with strain effects [65].

#### 3.1.2. Interfacial Perimeter

The interfacial region around the NPs forms a unique environment as the NPs, support and reactants make direct contact and synchronously promote the catalytic reaction. As a result, it can significantly enhance the adsorption and reactions of molecules at the perimeter [66]. The close proximity of NPs to different groups or defects (e.g., oxygen vacancies, hydroxyl groups, Lewis acids or Lewis bases) on the surface of the support may also contribute to localized sequential reactions of reactants or products, or the stabilization of transition states [45]. While this phenomenon of MSI can modulate the intensity of oxygenated intermediates adsorption by the catalyst, it accelerates the kinetics of the catalyst’s reaction to the OER process. Chen et al. rationalised the design and synthesis of RuCoO_x_ -RuCo-NC using a cobalt-based metal-organic backbone (MIL-L-Co) with a CoRu-based metal-oxide heterostructure. The catalyst showed excellent electrocatalytic performance in acidic environments due to the synergistic effect of spatial confinement and the interfacial effect of MSI. A current density of 10 mA cm^−2^ can be achieved with an overpotential of 228 mV, and excellent long-term durability is shown within 12 h of operation [67].

#### 3.1.3. Nanoparticle Morphology

The shape and crystal structure of NPs have a strong influence on their catalytic performance. This is because NPs of different morphologies expose distinct crystalline facets, which can result in either favorable or unfavorable atomic configurations in different reactions. Therefore, the activity and selectivity of catalysts can be effectively modulated by modulating the morphology of NPs at the metal-support interface [68]. Supported iridium- and ruthenium-based NPs are potential OER catalysts for PEMWE, but the inhomogeneous dispersion of these NPs on the support and their inhomogeneous size usually leads to migration and adhesion of the catalyst at high potentials and strong acidity. Ultimately, this leads to a loss of active surface area and catalytic material, resulting in reduced OER performance. To circumvent such problems, Xu et al. reported a surface atom step-enriched ruthenium-iridium (RuIr) nanocrystal uniformly dispersed on a metal-organic backbone (MOF)-derived carbon support (RuIr @ CoNC). The impressive performance of RuIr @ CoNC has been demonstrated both experimentally and computationally. Due to the presence of a large number of atomic steps that maximized the exposure of the catalytic active site and reduced the limiting potential of the catalyst, the catalyst exhibited excellent OER performance, showing an ultra-high mass activity of 2041 A gRuIr^−1^ at an overpotential of 300 mV. The strong MSI between the RuIr nanocrystals and the CoNC support gave the RuIr catalyst the ability to be uniformly dispersed and firmly anchored on the CoNC, conferring outstanding stability to the catalyst, which was maintained at 10 mA cm^−2^ for 40 h without significant decay [69].

#### 3.1.4. Chemical Composition

**Figure 2:**
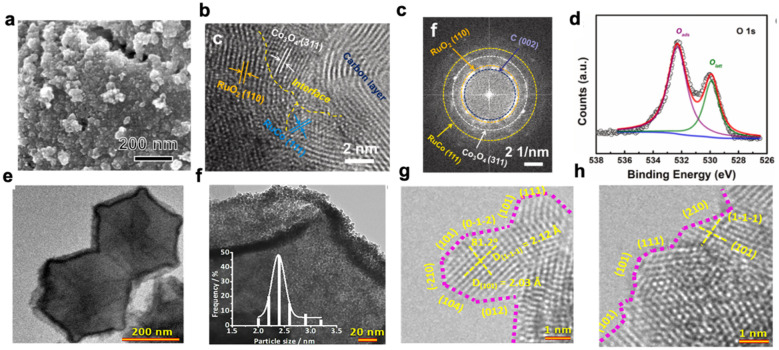
(**a**) SEM images of RuCoO_x_-RuCo-NC; (**b**) line profiles of RuCoO_x_-RuCo-NC; (**c**) HAADF-STEM elemental mappings; (**d**) XPS spectra of RuCoO_x_-RuCo-NC: O 1s [66]; (**e**) SEM image of RuIr@CoNC catalysts; (**f**) size distribution of RuIr nanocrystals; (**g,h**) HRTEM images. Atomic steps are indicated with pink dotted lines [69].

Solid-state reactions can occur between the metal NPs and the support, resulting in the formation of a new phase. The exchange of species is possible in both directions and is usually designed for redox reactions [68]. The local composition of alloyed metal NPs can be affected by interactions with the support, where the rearrangement of components in alloyed NPs is driven by the interaction of their elements with the support at the interface. This can lead to compositional rearrangements that differ from the initial homogeneous composition, such as sub-nanostructures like core-shells, affecting the synergy between the metal and metal active sites and thus, their catalytic performance (Figure 2) [70].

### 3.2. Strong Metal-Support Interaction (SMSI)

Although SMSI is also an MSI phenomenon, because it has special characteristics compared to the other four common MSI phenomena, it will be discussed separately. SMSI was first proposed by Tauster et al. in 1978 [70]. Since then, researchers have conducted in-depth studies on the formation mechanism of SMSI, and it is generally agreed that one of the main reasons for the formation of SMSI is the different surface energy and work functions between metal NPs and supports, which drive the surface migration of the supports to the metal NPs, thus stabilizing the metal NPs [68]. SMSIs are normally differentiated according to the different construction methods. SMSIs constructed using high temperature reduction are referred to as classical SMSIs [70]; those constructed with an oxygen atmosphere are referred to as O-SMSIs [71]; and those formed using adsorbent induction are referred to as A-SMSIs [72]. Although there are various methods of SMSI construction, there is still a lack of effective methods to purposefully construct SMSI on some relatively inert oxide supports such as SiO_2_, CaO, Al_2_O_3_ and so on [73]. The use of SMSI can effectively modulate the distribution, valence, and ligand structure and stability of NPs on the surface of the support, creating great opportunities for the electronic structure modulation of the active component of the catalyst [73]. Xu et al. synthesized a series of M-Ru/RuO_2_@CNT (M = Mn, Cd, Cu) bifunctional OER/HER catalysts. The OER activity and stability of the catalysts were improved by strong SMSI. The optimized Mn-Ru/RuO_2_@CNT catalyst with an ultra-small particle size of 2.5 nm showed the best catalytic performance, with a low overpotential of 177 mV and 30 mV for OER and HER, respectively, at a current density of 10 mA cm^−2^ in 0.5 M H_2_SO_4_ [74].

Specifically, catalysts with SMSI have the following characteristics, which may be utilized to enhance the catalytic activity of the catalysts.

**Figure 3:**
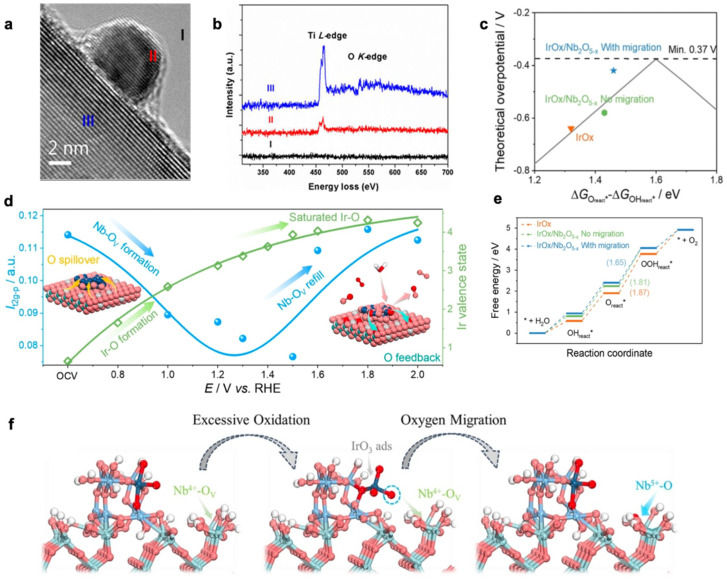
(**a,b**) EELS spectra of the Au/TiO_2_ sample. Spectra were background-subtracted. In-situ STEM images of sintered 6% Rh/TiO_2_ after treatment in 5% H_2_ and 95% N_2_ at 550 °C for 10 min [75]. (**c**) Gibbs free energy diagram for AEM on IrO*_x_*, IrO*_x_*/Nb_2_O_5-*x*_ (no migration) and IrO*_x_*/Nb_2_O_5-*x*_ (with migration). The dashed line with the arrow indicates the Δ*G* of the potential-determine step. (**d**) Illustration of the overall dynamic interface effect. The left Y axis colored in blue shows the variation of 4d_t2g_-p peak intensity, which is indicative of the oxygen vacancy content. The right Y axis colored in green shows the variation of Ir valence state. The color scheme for the chemical representation: cyan, Nb; blue, Ir; red, O; silver, oxygen vacancy. (**e**) Theoretical overpotential as a function of the OER activity descriptor characterized by the adsorption free energy difference between O_react_* and OH_react_*. (**f**) Proposed over-oxidation and recovering pathway for Ir on Ir/Nb_2_O_5-x_ considering the dynamic migration of oxygen species. The blue dashed circle in (**f**) is implying the oxygen atom that undergoes migration to fill in Nb^4+^ -OV. The color scheme for the chemical representation: cyan, Nb; blue, Ir; red, O; white, H [76].

(1) The migration of the support to the surface of the metal particles to form an inclusion structure. Zhang et al. constructed a classical SMSI by high temperature treatment of Au/TiO_2_. The presence of an amorphous inclusion layer was observed by HETEM and the chemical components of the inclusion layer were analyzed by EELS (Figure 3a,b) [75]. The inclusion layer acted as immobilization and protection for the NPs. In the A-SMSI constructed in Rh/TiO_2_, the Rh NPs were wrapped by amorphous TiO_x_ and the inclusions were composed of Ti species of different valence states (Ti^3+^/Ti^4+^=3/7), whereas in the Rh/TiO_2_ catalyst with classical SMSI effect, the Rh NPs were wrapped by a more crystalline TiO_x_ diatomic layer and only Ti^3+^ were present in the inclusions [72]. These studies suggest that the structure and composition of the inclusions on the surface of metal NPs can be tuned by different SMSI construction methods. Shi et al. reported on an Ir/Nb_2_O_5-x_ catalyst and the role of the catalyst/support interface in the catalytic reaction process was investigated. Characterization demonstrated an increase in the oxygen vacancy (O_V_) content of the support, which was in agreement with a decrease in the Nb valence state. This suggests that O on the support may have migrated during the reaction. Further, an isotopic labelling method was used to isotopically label the supports with ^18^O before loading them with Ir NPs. In-situ electrochemical mass spectrometry analysis showed an increased ratio of ^18^O/^16^O in the OER product, indicating migration of ^18^O from the support to Ir. At high oxidative potentials, the oxygen vacancy content of the support decreased and the valence state of Nb increased, indicating migration of over-coordinated oxygen species from Ir to the support, is beneficial to inhibit the deep oxidation of Ir. These experimental characterization results suggest that the nature of the catalyst/support interface effect is the dynamic migration of oxygen species between the support and the active species. As is shown in Figure 3e,f, DFT calculations show that OH_react_* migrates from the Ir site to the Nb site as the reaction proceeds, and when OOH _react_* is adsorbed, OH_react_* migrates back to the Ir site. The reaction energy for Ir/Nb_2_O_5-x_-catalyzed OER is 1.81 eV when OH migration is not considered and is reduced to 1.65 eV when OH migration is considered. The optimized Ir/Nb_2_O_5-x_ expresses excellent catalytic performance in PEMWE devices. The electrolytic voltage was only 1.839 V at a high current density of 3 A cm^−2^ and the cell could be operated stably for 2000 h at a current density of 2 A cm^−2^ without significant decay [76].

(2) Intermetallic bonding. Tauster et al. suggested that intermetallic bonding was facilitated by the overlap of the d and empty d orbitals of the excess metal between the active component and the support [70]. An Au/ZnO catalyst reported by Chung et al. showed that Au-Zn interactions could be formed by treating Au/ZnO with oxygen at different temperatures by fine structure resolution of Au by EXAFS. The formation of Au-Zn bonds (similar to AuZn alloys) can be directly detected after reduction at 300 °C under hydrogen atmosphere [71]. The phenomenon of intermetallic bonding caused by SMSI can be a rational strategy for researchers to regulate the electronic structure of active components in OER catalysts. In response to the limitations of the conventional AEM and LOM evolutionary mechanisms on the performance as well as the stability of acidic OER catalysts, Lin et al. innovatively reported an OER electrocatalyst (Ru/MnO_2_) with Ru atomic array patches supported on α-MnO_2_. The OER catalytic mechanism of this catalyst involves only *O and *OH species as intermediates. This mechanism allows the direct coupling of O-O radicals to generate and precipitate O_2_. Ru/MnO_2_ exhibits high OER activity (161 mV@10 mA cm^−2^) and excellent stability (minimal loss of activity after 200 h of operation) and is one of the best-performing catalysts available for acid-stable oxygen precipitation reactions. Extensive in-situ/ex-situ characterization as well as theoretical calculations demonstrate that the Ru species exhibits a lower oxidation state (<4) due to the electron-withdrawal effect of Mn in the lattice. OER on Ru/MnO_2_ proceeds via oxidation pathway mechanism (OPM), where the key step involves direct coupling of O-O radicals. This unique reaction pathway allows Ru/MnO_2_ to overcome the overpotential limitations of the conventional AEM mechanism. The dynamic cation exchange reaction between Ru ions and MnO_2_ during OER not only triggers the self-reconfiguration of the electrocatalyst, but also ensures that leached Ru ions can be recaptured to support further reactions, thereby enhancing corrosion resistance [77]. This work exploits the SMSI effect to explore new OER mechanisms and suggests new ideas for the design of OER catalysts from a mechanistic perspective.

(3) Modification of the adsorption characteristics of the catalytical active component. As early as 1982, Burch et al. found that the adsorption of H_2_ by the catalyst material was substantially reduced after high temperature reduction of Ni/TiO_2_ at 650 °C [78]. It has also been mentioned in the literature that O-SMSI and A-SMSI constructed by oxygen treatment and adsorbent induction lead to a significant reduction in the adsorption capacity of the material for small molecules such as CO and H_2_ [72,79]. The property that SMSI can change the adsorption characteristics of the material allows one to design SMSI to adjust the adsorption energy of the catalyst material for OER reactions on intermediates, thus improving the catalytic effect of the catalyst and even improving the stability of the catalyst. According to the OER volcanogram, tungsten oxide has a relatively weaker O-binding capacity than iridium oxide, and it is thus possible to coordinate the O-binding capacity by constructing a W-Ir dual active site on the catalyst [80]. However, the actual catalytic effect of tungsten oxide in acidic OER is not satisfactory, due to its degradation by intermediates in the OER reaction [81]. Lu et al. constructed Ir-W@Ir-WO_3-x_ core-shell structure catalysts with an Ir-W metal core and an Ir-doped WO3x shell (Ir-WO_3-x)_ by thermal reduction of Ir-W@Ir-WO_3-x_. It was further found that the moderate adsorption strength of Ir-W@Ir WO_3-x_ on oxygen-containing intermediates not only accelerated the oxygen evolution reaction, but also inhibited the formation of hydrogen peroxide, resulting in excellent catalytic activity and stability to acidity. The charge interaction between O and Ir on the Ir_sub_-W@Ir-WO_3_-O_v_(110) surface is stronger than the W-O interaction in WO_3_(001), but weaker than the Ir-O interaction in Ir-WO_3_(001), which leads to a moderate adsorption free energy for O on the Ir_sub_-W@Ir-WO_3_-O_v_(110) surface, and the moderate adsorption capacity can be determined by an equilibrium potential step which confers excellent catalytic activity. The catalyst achieved a low overpotential of 261 mV at 10 mA cm^−2^ and only a 3.5% potential rise in a 20 h chrono test, providing good stability that exceeds most previously reported acidic OER catalysts [82]. This work improves the OER activity and stability of the catalysts by constructing the SMSI of Ir-W with Ir-WO_3-x_, demonstrating the potential applications of SMSI for acidic OER catalyst design.

(4) The SMSI phenomenon is reversible. Under certain conditions, the above three features of SMSI can be eliminated or reconstructed again. Braunschweig et al. discovered through electron microscopy that Rh wrapping layers in Rh/TiO_2_ catalytic materials can be reconstructed and faded away under high-temperature reduction-oxidation conditions [83]. The reversible nature of SMSI gives researchers more room for manipulation and experimentation.

### 3.3. Strong Oxide-Support Interaction (SOSI)

Initially, the SMSI was primarily concerned with strong interactions between metal atoms and supports, but this was gradually extended to strong interactions between metal-containing species and supports [84]. The concept of SOSI was derived to better describe the strong interactions between oxides and supports in order to distinguish them from SMSI. In general, similar to those of SMSI, SOSI has the following effects.

**Figure 4:**
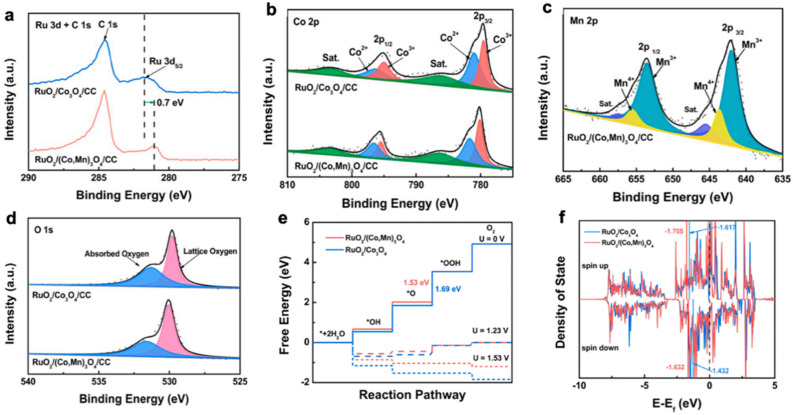
XPS spectra of (**a**) Ru 3d, (**b**) Co 2p, (**c**) Mn 2p, and (**d**) O 1s of RuO_2_/Co_3_O_4_/CC and RuO_2_/(Co, Mn)_3_O_4_/CC samples. (**e**) Gibbs free energy diagrams of supported catalysts. (**f**) Projected density of states (PDOS) of Ru d orbitals in supported catalysts [85].

**Figure 5:**
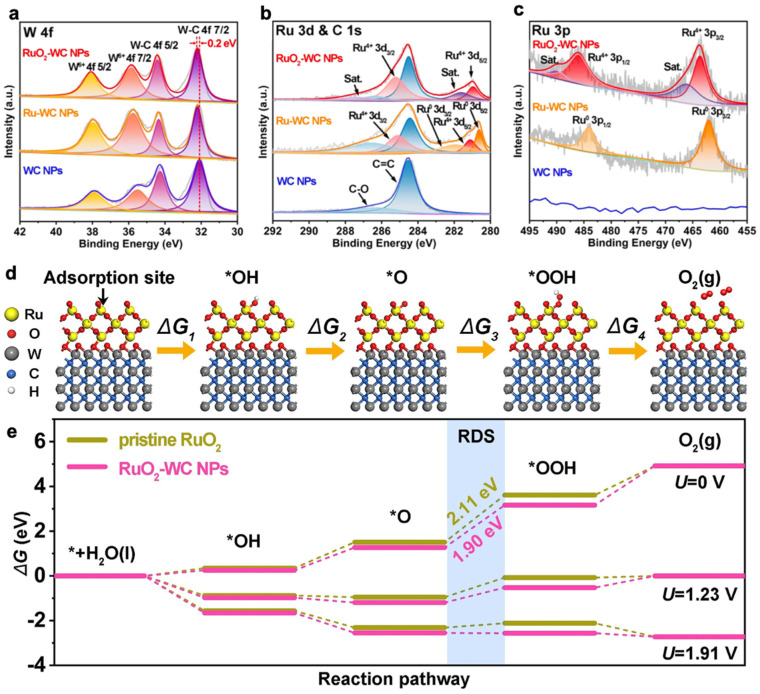
XPS spectra of RuO_2_–WC NPs, Ru–WC NPs and WC NPs. High-resolution XPS spectra of (**a**) W 4f, (**b**) Ru 3d and C 1s, and (**c**) Ru 3p. (**d**) Mechanism of OER process steps and corresponding Gibbs free energies of reaction intermediates on RuO_2_–WC NPs. (**e**) Free energy landscape of pristine RuO_2_ and RuO_2_–WC NPs at zero potential (*U* = 0), equilibrium potential (*U* = 1.23 V), and the potential (*U* = 1.91 V) for which each step is downhill of RuO_2_–WC NPs, respectively [86].

(1) SOSI can redistribute electrons at the oxide-support interface and modulate the electronic properties of the catalytic active site. Niu et al. reported the synthesis of a RuO_2_/(Co, Mn)_3_O_4_ nanocomposite. The introduction of manganese in Co_3_O_4_ resulted in the redistribution of support electrons. By XPS characterization, the authors found that RuO_2_/(Co, Mn)_3_O_4_ catalysts appeared as electron-rich Ru species. The adsorption of O on RuO_2_/(Co, Mn)_3_O_4_ was weakened as the electronic properties changed, which resulted in the rate-determining step in the OER process, i.e., the formation of OOH*, being accelerated. This cost-effective catalyst (ultra-low Ru content of 2.51 wt%) requires an overpotential of only 270 mV to achieve a current density of 10 mA cm^−2^ with a mass activity nearly 69 times better than that of commercial RuO_2_ (Figure 4) [85].

(2) SOSI can also effectively reduce the aggregation of NPs in electrocatalytic reactions, thereby improving the atomic utilization and stability of the material. WC-loaded RuO_2_ NPs (RuO_2_-WC NPs) anchored on carbon nanosheets reported by Sun et al. exhibited strong catalyst-support interactions and the low loading of Ru (4.11 wt.%) significantly enhanced the acidic oxygen precipitation reaction activity. The 10 mA/cm^2^ overpotential was 347 mV and the mass activity was eight times higher than that of commercial RuO_2_. The excellent OER performance can be attributed to the catalyst-support interaction between RuO_2_ and the WC support. Theoretical calculations show that the strong catalyst-support interaction between RuO_2_ and WC supports optimizes the electronic structure around the Ru site, giving good adsorption energy for OER intermediates and thus reducing the reaction potential of the RDS. At the same time, the WC supports contribute more electrons to the catalyst surface to protect the Ru active site from excessive oxidation during the acidic OER process. Notably, WC supports can reduce the amount of RuO_2_ added, resulting in higher Ru utilization than commercial RuO_2_ and other Ru-based electrocatalysts [86].

(3) SOSI can improve the catalytic performance of OER catalytic materials by building rich interfaces, thus improving the catalytic performance of OER catalytic materials. Highly dispersed IrO_2_ nanoclusters (~1 nm) loaded on porous V_2_O_5_ supports were successfully prepared by Zheng et al. using a MIL-88B(V) organometallic framework as a self-sacrificing template. Through detailed characterization and comparative experiments, the strong interaction between IrO_2_ nanoclusters and V_2_O_5_ supports resulted in significant lattice distortion of IrO_2_ nanoclusters, which facilitated the exposure of more unsaturated coordination active sites. At the same time, the strong electron transfer (electron transfer from Ir to V) at the interface between IrO_2_ and V_2_O_5_ allows the IrO_2_ active sites to act as electrophilic centers, weakening the adsorption of oxygen-containing intermediates. DFT calculations suggested that the SOSI effect alters the occupation number of Ir eg orbitals, lowering the *O to *OOH energy barrier and thus effectively promoting the reaction rate of water oxidation. As a result, the IrO_2_/V_2_O_5_ catalyst exhibited excellent OER activity in different media, exhibiting not only an ultra-low OER overpotential of 266 mV at 10 mA/cm^2^ but also a high-performance all-water decomposition over a wide pH range (Figure 5) [87].

### 3.4. Electron-Metal-Support Interactions (EMSI)

EMSI can lead to changes in the electronic structure of the metal particles and alter the adsorption and activation energies of reactant molecules, resulting in enhanced catalytic properties. In 2012, Campbell et al. first proposed electronic metal-support interactions (EMSI) based on the interaction between platinum and cerium atoms [88]. Specifically, EMSI refers to the charge redistribution phenomenon at the interface between the two, the support and the NPs, in a loaded metal nanoparticle catalyst. This is manifested as a local environmental change in the metal site, causing a change in the d-band (ε_d_) structure of the metal. By designing and modulating EMSI, the ε_d_ of the metal can be increased, thereby enhancing the catalytic performance of the catalyst [84,88]. Bruix et al. loaded Pt NPs onto CeO_2_ and found that the catalyst showed a significant improvement in catalytic performance in water gas conversion reactions. The team used EMSI to explain this phenomenon. By correlating the chemisorption energy with the electronic properties of the metal (i.e., the d-band centre), the description of its electronic state confirms the existence of EMSI and explains the enhanced catalytic activity associated with the metal-support interface [89].

Catalyst systems with typical EMSI effects have been identified: metal-doped carbon (M-dN), metal-transition metal compound groups (M-TMCs) and metal-metal substrates [84]. EMSI involves the formation of chemical bonds due to the mixing of metal d-orbitals. On the one hand, this interaction stabilizes the metal NPs; on the other hand, the support effect will greatly influence the catalytic performance of the metal NPs [90,91].The energy balance at the metal-support interface reduces the activation energy of the reaction and thus increases the catalytic activity of the catalyst. The electron flow at the metal-support interface is decisive for the Fermi energy balance of the catalyst [92]. The properties of the support, the loaded metal NPs, defects, and so on can influence the electron transfer process [64]. The net electron transfer across the metal-support interface enhances the chemisorption of the metal NPs to the reactants [93]. Shi et al. modulated the electronic structure of MoS_2_-loaded Pd NPs by using EMSI and investigated the effect of catalyst-support interactions on the catalytic activity of the reaction for the degradation of methylene blue (MB) using the electron donor NaBH_4_. Mechanistic studies showed that electrons are transferred from Pd to the MoS_2_ surface and a highly electron-deficient state is formed on the Pd surface. This electron structure makes the Pd surface favorable for the adsorption of electron-rich reactants during the catalytic process, thus accelerating the transfer of electrons from NaBH_4_ to MB [94].

EMSI provides a more detailed explanation of the enhanced catalytic performance of catalyst-support interactions than MSI, SMSI and SOSI, pushing the interpretation of the catalytic effect to the electronic scale. However, systematic theoretical and operational experiments have rarely been reported. As accurate identification of the electronic state is often difficult or even impossible for metal particles or clusters. However, as single-atom metal catalysts have entered the limelight, the inherent metal effects can be avoided as the supported metal species are narrowed down to single atoms and the interaction between the active site and the support is always homogeneous, which can be more easily characterized by experimental and theoretical calculations for EMSI.

In the field of acidic OER, a more detailed study of EMSI between metal atoms was carried out by Wu et al. They constructed a series of PtCux/Pt_skin_ core-shell structures with a single Ru atom. As the XAS results show, an increase in WL was observed on the Pt L3 side as the potential was scanned from 0 to 1.86 V, suggesting that the increase in the d-band vacancies in Pt may be due to electrons being transferred to Ru. During the voltage scan, Ru appears to be in a stable valence state due to the transfer of electrons from Pt-Cu to Ru. EMSI between Ru and Pt-Cu prevents Ru from being over-oxidized. Due to the proper oxidation state, the OER on Ru proceeds in the AEM mechanism, as evidenced by the peak corresponding to OOH at 1212 cm^−1^ in the in-situ attenuated total reflection infrared spectroscopy-electrochemical measurements. The calculations further demonstrate that EMSI between Ru and Pt-Cu not only stabilizes the Ru atoms, but also accelerates the electron transfer and promotes the catalytic efficiency of the catalyst for OER [95]. This work illustrates the positive impact of EMSI on the catalytic performance of loaded catalysts and gives a method to optimize the performance of metal/oxide catalysts, which is an important reference for the design of new acidic OER catalysts.

Kern et al. prepared a series of bimetallic catalysts based on a two-dimensional MOF. They found that the increase in catalytic activity was not linearly related to the increase in the number of active centers, which may be due to a synergistic effect between the two metal centers resulting in a change in electronic structure. Furthermore, XAS showed a strong magnetic coupling between Fe and Co, and π-electrons may form EMSI between Fe, Co and the network, promoting surface transition effects and achieving high activity towards OER [63].

### 3.5. Comparison of Different Catalyst-Support Interactions

In terms of comparing and distinguishing these interactions, it is worth noting that SMSI and SOSI are both specific types of metal-support interactions, with the former being observed in noble metal catalysts supported on reducible metal oxides, and the latter being observed in transition metal catalysts supported on oxides. Both SMSI and SOSI can lead to changes in the electronic and chemical properties of the metal particles, resulting in enhanced catalytic properties. MSI, on the other hand, is a more general term that encompasses all metal-support interactions.

EMSI is a separate type of interaction that is based on the electronic structure of the support material rather than its chemical properties. EMSI can influence the electronic structure of the metal particles, leading to changes in the catalytic properties of the catalyst. Compared to SMSI and SOSI, EMSI is a more subtle effect that can be difficult to observe and quantify.

## 4. Conclusions and Discussion

Here, we summarize common catalyst-support interactions that can potentially be used to improve the catalytic performance and stability of OER catalysts used in acidic media. Although the catalyst-support interactions utilized vary, the ultimate aim is to adapt the atomic arrangement structure of the catalyst to the dominant mode of reactant absorption/desorption and to match the electron orbital energy levels to those of the reactant molecules, which in turn greatly enhances the catalytic performance of OER in acidic media. At the same time, through the catalyst-support interaction, we can modulate the nanostructure of the NPs, using the metal-support interaction to firmly anchor the metal nano atoms to the support, and modulate the electronic structure of the NPs to prevent their over-oxidation and leaching, thus improving the stability of the catalyst, which is of general significance for the modification of OER catalysts used in acidic media.

In summary, the unique role of catalyst-support interactions such as MSI, SMSI, SOSI and EMSI in modulating the structure and performance of acidic OER catalysts is highly dependent on the nature of the metal and support, as well as the preparation conditions. Understanding these interactions and their effects on the catalyst is crucial for the design of more active and stable acidic OER catalysts.

However, there is still a lack of clear demarcation between the various catalyst-support interactions. More in-depth studies are needed to define and differentiate between the various interactions to provide a clearer methodology for the use of catalyst-support interactions to modulate catalyst performance and stability. At the same time, there is still a need to develop characterization tools for catalyst-support interactions, which are important for understanding the mechanisms of catalyst-support modulation of catalyst geometry and electronic structure at the periplasmic level.

While many catalysts currently show good activity and stability in half-cells, there is a lack of practical testing of PEMWE systems, which require a significant increase in voltage for large-scale application. Furthermore, the highly acidic environment of MEA remains a challenge for OER catalyst stability. To address these challenges, future research on acidic OER catalysts for PEMWE will focus on developing cost-effective and earth-abundant materials, optimizing catalyst-support interactions, and designing bifunctional catalysts. Advanced characterization techniques and computational modeling will guide the rational design of new materials with improved activity and stability. In addition, to develop cost-effective, high-performance, and highly stable acidic OER catalysts for PEMWE, it will be necessary to design nano-microstructures and improve mass transfer in a synergistic manner. This development is crucial to enable large-scale production of hydrogen as a clean and sustainable energy carrier and to promote the widespread adoption of PEMFCs technology.
